# The risk factors associated with COVID-19-Related death among patients with end‐stage renal disease

**DOI:** 10.1186/s12882-020-02221-w

**Published:** 2021-01-19

**Authors:** Hadith Rastad, Hanieh-Sadat Ejtahed, Gita Shafiee, Anis Safari, Ehsan Shahrestanaki, Zeinab Khodaparast, Neda Shafiabadi Hassani, Mohammad Rezaei, Morteza Nazari, Akram Zakani, Mohammad Mahdi Niksima, Mehdi Azimzadeh, Fatemeh Karimi, Ramin Tajbakhsh, Mostafa Qorbani

**Affiliations:** 1 Social Determinants of Health Research Center, Alborz University of Medical Sciences, Karaj, Iran; 2 Endocrinology and Metabolism Research Center, Endocrinology and Metabolism Clinical Sciences Institute, Tehran University of Medical Sciences, Tehran, Iran; 3 Obesity and Eating Habits Research Center, Endocrinology and Metabolism Clinical Sciences Institute, Tehran University of Medical Sciences, Tehran, Iran; 4 Chronic Diseases Research Center, Endocrinology and Metabolism Population Sciences Institute, Tehran University of Medical Sciences, Tehran, Iran; 5 Student Research Committee, Alborz University of Medical Sciences, Karaj, Iran; 6 Non-communicable Diseases Research Center, Alborz University of Medical Sciences, Karaj, Iran; 7 Clinical Research Development Center of Kamali hospital, Alborz University of Medical Sciences, Karaj, Iran; 8 Cardiovascular Research Center of Rajaei, Alborz University of Medical Sciences, Karaj, Iran; 9 Clinical Research Development Center of Rajaei, Alborz University of Medical Sciences, Karaj, Iran; 10 Dietary Supplements and Probiotic Research Center, Alborz University of Medical Sciences, Karaj, Iran

**Keywords:** COVID-19, Death, End‐stage renal disease, Kidney failure diseases, Risk factors

## Abstract

**Background:**

The extent to which patients with End-stage renal disease (ESRD) are at a higher risk of COVID-19-related death is still unclear. Therefore, the aim of this study was to identify the ESRD patients at increased risk of COVID-19 -related death and its associated factors.

**Methods:**

This retrospective cohort study was conducted on 74 patients with ESRD and 446 patients without ESRD hospitalized for COVID-19 in Alborz province, Iran, from Feb 20 2020 to Apr 26 2020. Data on demographic factors, medical history, Covid-19- related symptoms, and blood tests were obtained from the medical records of patients with confirmed COVID-19. We fitted univariable and multivariable Cox regression models to assess the association of underlying condition ESRD with the COVID-19 in-hospital mortality. Results were presented as crude and adjusted Hazard Ratios (HRs) and 95% confidence intervals (CIs). In the ESRD subgroup, demographic factors, medical history, symptoms, and blood parameters on the admission of survivors were compared with non-survivors to identify factors that might predict a high risk of mortality.

**Results:**

COVID-19 patients with ESRD had in-hospital mortality of 37.8% compared to 11.9% for those without ESRD (*P* value < 0.001). After adjusting for confounding factors, age, sex, and comorbidities, ESRD patients were more likely to experience in-hospital mortality compared to non-ESRD patients (Adjusted HR (95% CI): 2.59 (1.55–4.32)). The Log-rank test revealed that there was a significant difference between the ESRD and non-ESRD groups in terms of the survival distribution (χ2 (1) = 21.18, *P-*value < 0.001). In the ESRD subgroup, compared to survivors, non-survivors were older, and more likely to present with lack of consciousness or O2 saturation less than 93%; they also had lower lymphocyte but higher neutrophil counts and AST concentration at the presentation (all *p* –values < 0.05).

**Conclusions:**

Our findings suggested that the presence of ESRD would be regarded as an important risk factor for mortality in COVID-19 patients, especially in those who are older than age 65 years and presented with a lack of consciousness or O2 saturation less than 93%.

## Background

The Coronavirus disease 2019 (COVID-19) has rapidly spread in nearly three months after the discovery of its causative agent, severe acute respiratory syndrome coronavirus 2 (SARS-CoV-2), leading to the ongoing pandemic [1]. While males and females of all ages have presented with COVID-19, the majority of severe cases and fatalities have occurred in a specific proportion of patients, including the elderly and those with chronic underlying conditions up to now [2–4].

Patients with end-stage renal disease (ESRD) are more likely to develop COVID-19 than the general population due to a weak immune system and regular presence at the healthcare facilities [5, 6].On the other hand, these patients, who are often elderly with several underlying conditions, are supposed to be more susceptible to the poor outcomes of COVID-19 [7, 8].

Overall, ESRD has not been identified as a main prediction factor for poor outcomes in patients with COVID-19 in most previous studies [2, 9]. The lack of the presence of kidney failure diseases among the prognostic factors of COVID-19 may be misinterpreted that these patients are not mainly at a higher risk for the poor outcomes of COVID-19 [6]. In other words, there is still insufficient evidence regarding the prognostic impact of ESRD on COVID-19 patients [6]. Accordingly, the objectives of this study were to: 1) identify the extent to which ESRD patients are at a higher risk of COVID-19 in-hospital mortality and (2) determine the risk factors associated with the COVID-19 in-hospital mortality in ESRD patients.

## Methods

### The study population

In this retrospective cohort, the medical records of 640 patients aged 18 years or older hospitalized for COVID-19 in Alborz province, Iran, from Feb 20, 2020, to Apr 26, 2020, were assessed for ESRD status. Among all assessed medical records, a total of 74 patients were ESRD, 446 patients were without ESRD, and the ESRD status in 120 patients was unclear. We excluded patients with unknown ESRD status (120 patients) from this study. Also, none of the patients without ESRD were with CKD.

### Data collection

Data on demographic factors, medical history, Covid-19- related symptoms, and laboratory tests were extracted from the medical records of inpatients with confirmed Covid-19 by trained researchers.

Blood parameters including the counts of white blood cell (WBC), neutrophils, and lymphocytes, serum concentrations of creatinine, lactate dehydrogenase (LDH), albumin, aspartate and alanine transaminases (AST, ALT), hemoglobin (Hb), Erythrocyte Sedimentation Rate (ESR), and prothrombin time (PT) of patients on the first day of hospital admission were measured with commercial kits.

In addition, the pharyngeal swab specimens of all patients were collected and examined in predetermined laboratories across the province to detect SARS-CoV-2 viral nucleic acid using real-time polymerase chain reaction (RT –PCR) assay.

### Diagnosis of COVID-19

In this study, COVID-19 diagnosis was confirmed using one of the following criteria: (1) a positive RT–PCR result or (2) a positive pulmonary abnormality on chest CT based on the radiological criteria of COVID-9 infection.

### Definition of variables and outcomes

ESRD was defined by self-report of receipt of hemodialysis, peritoneal dialysis, or being a candidate for renal transplant. A Glasgow scale (GCS) of 8 or less was considered as “lack of consciousness.” Comorbidities were determined based on the patient’s self-report on admission. Comorbidities were initially treated as a categorical variable (yes vs. no) and then classified based on the absolute number of them (with one, two, or ≥ three comorbidity (ies). Furthermore, we entered important comorbidities, including diabetes mellitus (DM), hypertension (HTN), and cardiovascular disease (CVD) as well as other diseases (such as osteoarthritis, cancers, immunodeficiency disorders, and chronic disease of blood, liver, or respiratory).

The end-point of our study was discharged as cured (survivors) or dead (non-survivors). Patients were discharged from the hospital if they met the following criteria: lack of fever for at least 72 h, clinical alleviation of respiratory symptoms, and improvement in pulmonary abnormalities on chest CT imaging.

### Statistical analyses

Demographic, clinical, and laboratory data in the study groups were summarized using descriptive statistics, mean (standard deviation (SD)) or median (interquartile range (IQR)) for continuous variables and frequency (percentage) for categorical variables. Characteristics of ESRD and non-ESRD groups as well as survivors and non-survivors in ESRD group, were compared using two-tailed t-tests, Mann–Whitney U tests, Fisher exact, or chi-square tests, as appropriate.

Univariable and multivariable Cox regression models were applied to assess the association of underlying condition ESRD with the COVID-19 in-hospital mortality of. Of the covariates assessed, those with a *P*-value ≤ 0.2 based on a univariate test were included in the multivariable Cox model as a potential confounder. Results were presented as crude and adjusted Hazard Ratios (HRs) and 95% confidence intervals (CIs).

A log-rank test was also performed to detect if there were differences in the survival distribution between ESRD and non-ESRD groups. In both groups, patients were right censored at discharge time from the hospital.

Statistical significance was defined as a two-tailed *P*-value < 0.05. All statistical analyses were performed using SPSS version19.0, (SPSS Chicago, IL, USA).

## Results

A total of 74 patients with ESRD and 446 patients without ESRD hospitalized for COVID-19 were included in this study. In the ESRD group, all patients were on hemodialysis at the time of admission; 12 (16.2%) of them were renal transplant candidates.

Table 1 presents the general characteristics and disease-related symptoms in the study groups. There was no statistically significant difference between the ESRD and non-ESRD groups in terms of age, but the proportion of males was significantly greater in the ESRD group. The most common complaints at the presentation in both groups were shortness of breath, followed by fever/cough, and tiredness. While comorbidities such as HTN, DM, and CVD were more frequent in the ESRD group than non-ESRD group (all *p*-value < 0.05), the most common underlying condition was HTN in both groups, accounting for over 55.4% in the ESRD group and 24.4% in non-ESRD group.

Patients with ESRD were more likely to present with a lack of consciousness (16.1% vs. 7.8%, *P*-Value = 0.032), and receive invasive mechanical ventilation (28.4% vs. 9.2%, *p*-value < 0.001) on admission than patients without ESRD. In the subgroup of survivors, no significant difference was observed between the ESRD and non-ESRD groups with respect to the length of the hospital stay (median (IQR), days: 2 (0–5) vs. 3 (1–6), respectively) and the time interval from the admission to the death (median (IQR), days: 6 (1–12) vs. 5 (1–13), respectively). (both *p*-values > 0.05) (Table 1).

Rate of in-hospital mortality was higher in COVID-19 patients with ESRD (37.8% (28)) vs. than those without ESRD (11.9% (53)) (*p*-value < 0.001).

**Table 1:** General characteristics and disease-related symptoms in patients with and without ESRD

Characteristics	ESRD *N* = 74	Non-ESRD *N* = 446	*P*-value
Age (year), Mean (SD)	63.2 (15.1)	60.1 (18.7)	0.167
Age ≥ 65 years, % (N)	52.7% (39)	42.8% (191)	0.113
Gender Male, % (N)	68.9% (51)	45.3% (202)	< 0.001
Symptoms, % (N)			
Caught	37.1% (27)	45.3% (202)	0.224
Fever	40.3% (30)	35.7% (159)	0.473
Shortness of breath	62.9% (46)	59.0% (263)	0.554
Tiredness	21.0 % (15)	26.2% (117)	0.373
Being Ventilated on admission% (N)	28.4% (21)	9.2% (41)	<0.001
O2 saturation <93% % (N)	56.8% (42)	53.6% (239)	0.612
Lack of consciousness	16.1% (12)	7.8% (35)	0.032
Comorbidities			
HTN	55.4% (41)	24.4% (109)	<0.001
DM	47.3% (35)	20.2% (90)	<0.001
CVD	29.7% (22)	17.3% (77)	0.011
Other comorbidities^a^	10.8% (10)	9.4% (42)	0.706
Number of comorbidities			
No comorbidity	21.6% (16)	56.5% (252)	<0.001
1	29.7% (22)	22.6% (101)	
2	33.8% (25)	13.2% (59)	
≥ 3	14.9% (11)	7.6% (34)	
Length of the hospital stay (days), Median (IQR)	2 (0-5)	3 (1-6)	0.298
Time to the death (days) Median (IQR)	6 (1-12)	5 (1-13)	0.880

*DM* Diabetes Mellitus, *CVD *Cardiovascular disease, *ESRD *End-stage renal disease, *HTN *Hypertension, *IQR *Inter quartile range

^a^ Rheumatoid arthritis, cancer, immunodeficiency, and chronic disease of blood, liver, or respiratory system

Table 2 shows laboratory findings of admission in patients with and without ESRD. A higher neutrophil count (median (IQR): 7.9 (7.06–8.8) vs. 7.3 (6.3–8.1) ), ESR values (54.0 (26.0-74.5) vs. 35.5 (19.0–64.0)), serum creatinine (6.1 (4.5–8.4) vs. 1.0 (0.8–1.3)) and potassium concentrations (5.0 (4.4–5.3) vs. 4.2 (3.9–4.5)), a longer PT time (14.0 (13.5–16.0) vs. 13.5 (13.0–14.0)) was found in ESRD patients compared to non-ESRD patients, but a lower concentration of RBC (3.87 (3.16–4.55) vs. 4.63 (4.25–5.16)) and Hb (11.8 (9.4–13.7) vs. 13.1 (11.8–14.6)) was observed in ESRD patients compared to non-ESRD patients (all *P*-values < 0.05).

**Table 2:** Laboratory findings on admission and presence of the comorbidities in the study population by ESRD status

Characteristics	ESRD Median (IQR^a^)	Non- ESRD Median (IQR^a^)	*P*-value
WBC count, × 10^9^ per L,	9.4 (6.5–14.0)	8.1 (5.6–11.1)	0.058
Lymphocyte count,× 10^9^/L	1.51(0.98–2.23)	2.00 (1.21–2.80)	0.065
Neutrophils, ×10^9^/L	7.9 (7.06–8.8)	7.3 (6.3–8.1)	0.005
Hb, g/dL	11.8 (9.4–13.7)	13.1 (11.8–14.6)	0.008
RBC, million/mm3	3.87 (3.16–4.55)	4.63 (4.25–5.16)	< 0.001
Platelet counts, ×10^3^/Ml	188.5(139.5-240.5)	213.0 (165.0-264.5)	0.224
AST, U/L	32 (22–51)	34 (25–49)	0.897
ALT, U/L	27 (18–37)	26 (18–44)	0.569
Creatinine, mg/dl	6.1 (4.5–8.4)	1.0 (0.8–1.3)	< 0.001
PT, s	14.0 (13.5–16.0)	13.5 (13.0–14.0)	0.004
ESR, mm/h	54.0 (26.0-74.5)	35.5 (19.0–64.0)	< 0.009
CRP, mg/l	25.0 (4.0–75.0)	15.7 (3.1–61.5)	0.047
LDH, U/L	461 (374–732)	405 (329–549)	0.226
CPK, U/L	76.0 (45.0-237.0)	73.5 (14.3–135.7)	0.802
Sodium (Na), mmol/L	135 (130–139)	136 (133–138)	0.265
Potassium (K), mmol/L	5.0 (4.4–5.3)	4.2 (3.9–4.5)	< 0.001

*ESRD *End-Stage Renal Disease, *ALT *Alanine transaminases, *AST *Aspartate transaminases, *CRP *C-reactive protein, *CPK *Creatine phosphokinase, *ESR *Erythrocyte Sedimentation Rate, *Hb *Hemoglobin, *LDH *Lactate Dehydrogenase, *PT *Prothrombin Time, *RBC *Red Blood Cell, *WBC *White Blood Cell

^a^*IQR* Inter quartile range

Results from univariate Cox regression analysis showed that ESRD patients had a higher COVID-19 in-hospital mortality than non-ESRD patients (Crude HR (95% CI): 2.80 (1.76–4.43)). This finding was also observed in the multivariate Cox regression analysis after considering potential confounders age and sex, and DM, CVD, HTN, and other comorbidities (Rheumatism, cancer, immunodeficiency, and chronic disease of blood, liver, or respiratory system) (Adjusted HR (95% CI): 2.59 (1.55–4.32)) (Table 3).

**Table 3:** Prognostic impact of ESRD for the death of the COVID-19: COX Regression Analysis

Variable	Model I^a^	Model II ^b^	Model III ^c^
	Crude HR (95% CI)	Adjusted HR(95% CI)	Adjusted HR (95% CI)
**ESRD**	2.80 (1.76-4.43)	2.52 (1.57-4.04)	2.59 (1.55-4.32)

*HR* Hazard Ratio, *CI *Confidence Interval, *ESRD *End-Stage Renal Disease

^a^Crude model

^b^ Adjusted for age and sex

^c^ Adjusted for age and sex

Diabetes mellitus, cardiovascular disease, hypertension, and other comorbidities (Rheumatoid arthritis, cancer, immunodeficiency, and chronic disease of blood, liver, or respiratory system)

**Fig. 1:**
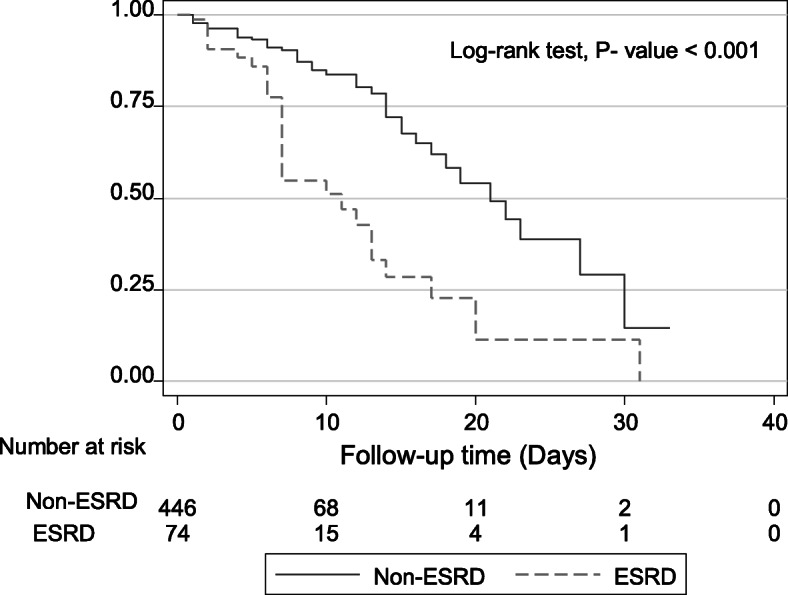
Survival curve in patients with COVID-19 by ESRD status (Kaplan Meier & log rank test)

Based on log-rank test results, a statistically significant difference was found between ESRD and non-ESRD groups in terms of the survival distribution (χ2 (1) = 21.18, *P*-value < 0.001) (Fig. 1).

In ESRD group, non-survivors were significantly older (mean (SD) age: 70.1 years (12.4) vs. 59.1 (15.3); *P* = 0.002). Non-survivors were more likely to present with lack of consciousness, have O2 saturation less than 93%, and receive invasive mechanical ventilation than survivors (all *p* –values < 0.05). The frequency of the comorbidities of HTN, DM, and CVD were similar between survivors and non-survivors. (all *p* –values > 0.05) Also, a lower lymphocyte count (median (IQR): 1.80 (1.00-2.50) vs. 1.20 (0.67–2.20), ×10^9^/L; *P*-value = 0.049) was found in non-survivors compared to survivors, but a higher neutrophil count (8.5 (7.8-9.0) vs. 7.5 (6.9–8.5), ×10^9^/L; *P*-value = 0.015) and AST concentration (44.0 (28.5–68.3) vs. 29.0 (20.6–39.0), U/L; *P*-value = 0.042) was observed in non-survivors compared to survivors. (Table 4)In univariate logistic regression model, age, Spo2 < 93%, lack of consciousness, and laboratory parameters, the count of WBC, lymphocytes, and neutrophils were significantly associated with COVID-19-related death in ESRD patients (All *p* values < 0.05). In the multivariate model, only po2 < 93% remained significant (OR: 6.63, *P*-value: 0.016)

**Table 4:** General characteristics and disease-related symptoms in ESRD patients by survival status

Characteristics	Survivors *N* = 46	Non-Survivors *N* = 28	*P*-value
Age (year), Mean (SD)	59.1 (15.3)	70.1 (12.4)	0.002
Age ≥ 65 years, % (N)	39.1% (18)	75% (21)	0.004
Gender Male, % (N)	60.9% (28)	82.1% (23)	0.072
Receiving invasive mechanical ventilation on admission% (N)	2.2% (1)	71.4% (20)	< 0.001
O2 saturation < 93% % (N)	41.3% (19)	82.1% (23)	0.001
Lack of consciousness	7.7% (4)	30.4% (8)	0.030
**Comorbidities**			
HTN	54.3% (25)	57.1% (16)	1.00
DM	41.3% (19)	57.1% (16)	0.233
CVD	26.1% (12)	35.7% (10)	0.437
Other comorbidities^a^	13.0% (6)	7.1% (2)	0.702
**Laboratory findings (Median (IQR))**			
WBC count, × 10^9^ per L,	9.4 (6.5–14)	9.6 (5.7–13.8)	0.735
Lymphocyte count,× 10^9^/L	1.80 (1.00-2.50)	1.20 (0.67–2.20)	0.049
Neutrophils, ×10^9^/L	7.5 (6.9–8.5)	8.5 (7.8-9.0)	0.015
AST, U/L	29.0 (20.6–39.0)	44.0 (28.5–68.3)	0.042
ALT, U/L	22.0 (15.5–37.0)	31.2 (23.7–37.0)	0.349
Creatinine, mg/dl	6.9 (4.8–9.1)	5.5 (4.3–7.6)	0.109
Hb, g/dL	10.7 (8.6–13.4)	12.3 (10.1–13.8)	0.190
RBC, million/mm3	3.8 (3.1–4.4)	3.9 (3.2–4.8)	0.919
MCV, fl.	87.9 (82.5–97.9)	86.6 (83.1–98.7)	1.00
MCH, pg	29.0 (27.3–31.0)	28.0 (26.8–32.1)	0.756
Platelet counts, ×10^3^/Ml	181.0 (125.5–248.0)	192.0 (163.5-236.5)	1.00
LDH, U/L	441.5 (342.5-591.7)	571.0 (410.0 -798.0)	0.695
ESR, mm/h	54.0(35.0–70.0)	48.0 (24.7–83.0)	0.867
CRP, mg/l	23.5 (3.0–75.0)	32.6 (15.0–79.0)	0.484
CPKk, U/L	68.5 (46.5-134.5)	136.0 (45.0-287.7)	0.372
Urea,	105.0 (92.0-183.0)	106.0 (24.0-157.0)	1.00
Sodium (Na), mmol/L	135.0 (128.8-137.5)	133.0 (130.0-142.5)	0.804
Potassium (K), mmol/L	5.1 (4.5–5.4)	4.8 (4.2–5.2)	0.134
PT, S	14.0 (13.5–16.2)	14.0 (13.5–15.5)	0.804

*IQR *Inter quartile range, *DM *Diabetes Mellitus, *HTN *Hypertension, *CVD *Cardiovascular Disease, *ESRD *End-Stage Renal Disease, *ALT *Alanine transaminases, *AST *Aspartate transaminases, *CRP *C-reactive protein, *CPK *Creatine phosphokinase, *ESR *Erythrocyte Sedimentation Rate, *Hb *Hemoglobin, *LDH *Lactate DeHydrogenase, *PT *Prothrombin Time, *RBC *Red Blood Cell, *WBC *White Blood Cell, *MCH *Mean Corpuscular Hemoglobin, *MCV *Mean Corpuscular Volume

^a^ rheumatoid arthritis, cancer, immunodeficiency, and chronic disease of blood, liver, or respiratory system

## Discussion

In this retrospective observational study, we investigated the prognostic impact of the ESRD- related Death in patients hospitalized with COVID-19. We showed that COVID-19 patients with ESRD had a higher in-hospital mortality rate than non-ESRD patients, even after adjusting for potential confounders, including age, sex, and comorbidities.

According to the results of a systematic review by Oyelade et al., the mortality rate was reported as 53.3% in CKD patients [10]. In the present study, the mortality rate in ESRD patients was 38%. Concordant with previous studies [11–14], the fatality rate of COVID-19 in ESRD patients was much higher than the general population. This could be partly explained by immune system dysfunction and high frequency of underlying comorbidities like hypertension, CVD, and diabetes in ESRD patients [15], although we observed the association between ESRD and high mortality even after adjusting for comorbidities. Generally, chronic kidney disease (CKD) is associated with an increased risk of pneumonia and a high pneumonia-related mortality rate [16, 17]. Moreover, the results of two recent meta-analyses revealed a significant association between preexisting CKD and severe COVID-19 [18, 19]. CKD has been associated with inflammatory status and impaired immune system [20]. Besides, as a result of over-expression of ACE2 receptor in the tubular cells of patients with CKD, the COVID-19 disease severity, and worse prognosis in these patients could be explained [21, 22].

In infected patients with ESRD, we compared clinical and laboratory characteristics between survivors and non-survivors to identify the risk factors associated with mortality. Non-survivors were significantly older, more likely to present lack of consciousness and O_2_ saturation less than 93%, and receive invasive mechanical ventilation than survivors; all these issues showed the worse general health in non-survivors. Moreover, non-survivors had lower lymphocyte count and higher neutrophil count and AST concentration compared to survivors. Abnormal liver enzyme concentration could be due to the systemic inflammation induced by the virus. Notably, older age is not specific for the ESRD patients, and it has been reported as a prognostic factor of death in the whole COVID-19 patients. In general, the prevalence of comorbidities is higher in older ages and the function of the immune system is weaker in this group [23, 24].

Consistent with previous findings [11], lymphopenia was a parameter associated with poor prognosis of COVID-19 disease in hemodialysis patients, which could be related to the disturbed immune function as an underlying mechanism. Goicoechea et al. conducted a study on hemodialysis patients of a single center in Spain and reported longer time on hemodialysis, low lymphocyte count, and high LDH levels as main predictors of mortality in infected patients [11]. In a report from four dialysis centers in Italy, the mortality rate of hospitalized COVID-19 patients was reported around 42%, near to our observation. Moreover, a high concentration of CRP as an inflammatory indicator and poor immune function was associated with death and a high concentration of AST was a risk factor for acute respiratory distress syndrome in these patients [14].

Comparison between the ESRD and non-ESRD groups showed that the proportion of males, frequency of lack of consciousness, and comorbidities, including HTN, DM, and CVD, and need to receive invasive mechanical ventilation were significantly higher in the ESRD group. It has been reported that the comorbidities associated with a high risk of COVID-19-related death are common in patients with CKD [14]. Notably, some symptoms of COVID-19 disease are not distinguishable from other symptoms common among patients on dialysis [25].

Regarding the laboratory findings of admission, patients with ESRD had a higher neutrophil count, ESR value, serum creatinine and potassium concentrations, longer PT time, while lower RBC and Hb compared to non-ESRD patients. Although previous reports declared that hemodialysis patients infected with COVID-19 presented less lymphopenia, the lower serum concentration of inflammatory cytokines, and milder clinical symptoms in comparison with other COVID-19 patients due to the uremia status [25, 26], we observed greater neutrophil count, CRP concentration and ESR value as well as lower lymphocyte count in the ESRD group.

This study has some limitations. First, due to the retrospective nature of the present study, we could not collect pre-hospital status including nutritional status and all laboratory tests for all patients evaluating their roles in in-hospital death. Second, we cannot rule out the confounding effects of the drugs which have been used for COVID-19 treatment during hospitalization as evidence suggests that some of the antiviral drugs may cause renal tubular dysfunction [27]. We also have no data regarding treatment regimens and use of antagonists on renin-angiotensin aldosterone system during the hospital stay of patients. Furthermore, some other non-host factors relevant to COVID-19 infection have been missed. Moreover, it should be noted that we gathered data regarding ESRD infected patients who needed hospital admission, and we have no view about the patients treated in the outpatient setting, which can affect the generalizability of results. So this issue should be taken into account when interpreting the results.

## Conclusions

The presence of ESRD would be regarded as an important risk factor for mortality in COVID-19 patients, and extra precautions should be taken by these patients. The most important clinical implication of this study is that physicians and healthcare providers should closely monitor and manage these vulnerable patients.

## Data Availability

The datasets used and/or analysed during the current study available from the corresponding author on reasonable request.

## Abbreviations

COVID-19: Coronavirus disease 2019
SARS-CoV-2: Severe acute respiratory syndrome coronavirus 2
ESRD: End-stage renal disease
WBC: White blood cell
LDH: Lactate dehydrogenase
AST: Aspartate transaminases
ALT: Alanine transaminases
Hb: Hemoglobin
ESR: Erythrocyte Sedimentation Rate
PT: Prothrombin time
RT–PCR: Real-time polymerase chain reaction
DM: Diabetes mellitus
HTN: Hypertension
CVD: Cardiovascular disease
SD: Standard deviation
IQR: Interquartile range
HRs: Hazard Ratios
